# Chitosan Increases Lysine Content through Amino Acid Transporters in *Flammulina filiformis*

**DOI:** 10.3390/foods11142163

**Published:** 2022-07-21

**Authors:** Li Tian, Zhaodi Ma, Hao Qiu, Xiaotian Liu, Tao Wu, Feng Ge, Rui Liu, Jing Zhu, Liang Shi, Ailiang Jiang, Hanshou Yu, Ang Ren

**Affiliations:** 1 Key Laboratory of Microbiological Engineering of Agricultural Environment, Ministry of Agriculture, Department of Microbiology, College of Life Sciences, Nanjing Agricultural University, Nanjing 210095, China; 2020116058@stu.njau.edu.cn (L.T.); 2018816153@njau.edu.cn (Z.M.); 2020816130@stu.njau.edu.cn (H.Q.); 2019116061@njau.edu.cn (X.L.); 2021116062@stu.njau.edu.cn (T.W.); 2021816129@stu.njau.edu.cn (F.G.); ruiliu@njau.edu.cn (R.L.); jingzhu@njau.edu.cn (J.Z.); shiliang@njau.edu.cn (L.S.); aljiang@njau.edu.cn (A.J.); yuhans@njau.edu.cn (H.Y.); 2 Institute of Biology, Guizhou Academy of Sciences, Guiyang 550009, China

**Keywords:** *Flammulina filiformis*, chitosan, lysine, amino acid transporter

## Abstract

**Highlights:**

- Chitosan treatment increased the lysine content of *F. filiformis*. Results from this study provide insights for industrial-scale trials potentially resulting in higher-quality *F. filiformis*.
- Response surface methodology (RSM) was used to optimize the conditions of chitosan-induced amino acid content in *F. filiformis* for the first time.
- The amino acid transporters in *F. filiformis* were systematically screened for the first time. The involvement of *AAT3* and *AAT4* genes in chitosan-induced lysine biosynthesis in *F. filiformis* was investigated by gene-silenced strains.

**Abstract:**

Lysine content is considered an important indicator of the quality of *Flammulina filiformis*. In this study, chitosan was used to improve lysine content of *F. filiformis*. Optimal design conditions were obtained using central combination design (CCD): treatment concentration was 14.61 μg/mL, treatment time was 52.90 h, and the theoretical value of lysine content was 30.95 mg/g. We used Basic Local Alignment Search Tool Protein (BLASTP) to search the *F. filiformis* genome database using known AATs in the NCBI database. There were 11 members of AAT in *F. filiformis*. The expression levels of *AAT3* and *AAT4* genes increased significantly with chitosan treatment. Subsequently, *AAT3* and *AAT4* silencing strains were constructed using RNAi technology. The lysine content of the wild-type (WT) strain treated with chitosan increased by 26.41%. Compared with the chitosan-induced WT strain, chitosan-induced lysine content decreased by approximately 24.87% in the *AAT3* silencing strain, and chitosan-induced lysine content in the *AAT4* silencing strain increased by approximately 13.55%. The results indicate that AAT3 and AAT4 are involved in the regulation of the biosynthesis of lysine induced by chitosan in *F. filiformis*. AAT3 may participate in the absorption of lysine, and AAT4 may be involved in the excretion of lysine with chitosan treatment.

## 1. Introduction

*Flammulina filiformis* (previously known as Asian *F. velutipes*) is one of the most popular mushrooms due to its good quality, low price and high nutrition [1,2,3]. It has been found that *F. filiformis* can reduce the serum total cholesterol level [4]. *F. filiformis* contains various beneficial components such as protein, amino acids and vitamins. Among them, amino acid content is rich, especially lysine, which is considered an important index reflecting the quality of *F. filiformis* [5,6]. Human and animals cannot synthesize lysine, so it must be obtained from food [7]. Overexpression of the saccharopine dehydrogenase gene improves lysine biosynthesis in *F. filiformis* [8]. Lysine can promote the absorption of protein in humans and animals [9]. With the improvement of living standards, people have higher and higher requirements for diet quality, and have gradually found that *F. filiformis* with its high nutrition and delicious taste is a good choice. Therefore, we need to cultivate well-growing *F. filiformis* with high lysine content under the conditions of factory culture and focus on studies that improve lysine content in *F. filiformis*.

Our predecessors have carried out extensive research on exogenous inducers to improve nutrient accumulation in edible fungi [10,11,12]. Studying the effects of exogenous inducers on the growth and metabolism of *F. filiformis* during production can increase the possibility of improving the quality of *F. filiformis*. It also provides practical guidance for the high-quality and high-yield cultivation of *F. filiformis*, which is of positive significance for the development of the *F. filiformis* industry. Nontoxic and pollution-free chitosan, which is widely used in medicine, food and environmental protection, has many biological functions such as antibacterial, anticancer and immunity enhancement [13,14]. In recent years, a large number of studies have also shown that chitosan has a broad prospect in agriculture. It can enhance the stress resistance of crops and can improve crop yield and the quality of agricultural products [15,16].

Amino acid transporters (AATs) play a key role in cell metabolism, not only transporting amino acids to the body for metabolism, but also participating in signal transmission [17]. At present, 24 AATs that mainly transport amino acids through the plasma membrane have been found in *Saccharomyces cerevisiae* [18]. Studies have shown that AATs, encoded by *AAT* genes, a key regulatory gene family in rice metabolism, play an important role in rice growth and development [19]. When overexpressing the proline transporter gene, damage to the strain caused by stress such as salt stress or drought stress can be reduced. In addition, the content of proline increased significantly in the overexpressed strains [20]. Overexpression of *AAP6* in rice can increase the content of amino acids and thus improve the quality [21]. However, it is still unknown whether AAT in *F. filiformis* can also regulate the content of specific amino acids.

Although *F. filiformis* have been artificially cultivated for a long time, the quality of *F. filiformis* has always been uneven. It is now necessary to improve quality as well as production [22]. Therefore, increasing lysine content in *F. filiformis* and studying the mechanism of its participation in AAT are important directions of *F. filiformis* research. In this study, the effect of chitosan on lysine content in *F. filiformis* was investigated. Based on the treatment concentration and timing of chitosan application, the optimal induction conditions of chitosan were explored by response surface methodology (RSM). The effect of chitosan on the fruiting body of *F. filiformis* was further investigated. Then, the AATs in *F. filiformis* were screened. The effects of AATs on the lysine content of *F. filiformis* induced by chitosan were investigated by constructing gene-silencing strains. This work provides a new idea for improving the quality of *F. filiformis*.

## 2. Materials and Methods

### 2.1. Strains, Culture Conditions and Induction Treatment with Chitosan of Mycelium of F. filiformis

*F. filiformis* strain Fv3 was obtained from the Research Institute of Shanghai Academy of Agricultural Sciences and inoculated on solid complete yeast medium (CYM). After culturing for 5 days in a constant-temperature incubator at 25 °C, ten pieces of mycelial blocks were aseptically picked and used to inoculate 100 mL of potato dextrose broth (PDB), then incubated in an oscillator at 25 °C and 150 rpm/min for 5 days. Subsequently, the liquid mycelium was aseptically broken evenly, and 3 mL was pipetted to 100 mL of CYM liquid medium. The culture was continued for 5 days in an oscillator at 25 °C and 150 rpm/min. After being cultured for 3 days, the same volume (100 μL) of different concentrations of chitosan were added, with the same volume (100 μL) of sterile water added to the control group, and for each group the procedure was repeated three times. The concentrations of chitosan were 0.01 mg/mL, 0.02 mg/mL and 0.03 mg/mL, respectively. The effect of chitosan incubation for different times (0-96 h) on lysine and protein content of *F. filiformis* was determined during a five-day period in CYM liquid medium.

### 2.2. Cultivation and Induction Treatment with Chitosan of Fruiting Body of F. filiformis

The cultivation of fruiting bodies was performed according to previously reported methods with appropriate modification [23]. Ingredients for cultivation: cotton seed shell 49%, corn cob 33%, wheat bran 6%, sugar 1% and gypsum 1%. *F. filiformis* was inoculated aseptically and cultured in the factory for about 55 days. After the *F. filiformis* overgrew the substrate (30 days), the old hyphae were removed. At this time, chitosan was used in the experiment. Chitosan was dissolved in deionized water, then sprayed on the surface of *F. filiformis* culture material after removing hyphae. Then, it still needed to be cultivated for about 25 days before harvesting. The concentrations of chitosan were 10 μg/mL, 14.61 μg/mL, 20 μg/mL and 30 μg/mL. Each bottle was used to spray 50 mL. Each treatment was repeated 9 times, and deionized water was used as control.

### 2.3. Determination of Mycelial Biomass, Growth of Fruiting Body, Total Protein and Lysine Content

The mycelium after fermentation was collected and dried in an oven at 60 °C. The weight of the dried mycelium was the mycelial biomass. Fruiting bodies were harvested and recorded during the first wave when their length was from 15 to 20 cm. Coomassie brilliant blue was used to determine the protein content in mycelium of *F. filiformis* [24,25]. A ninhydrin spectrophotometer was used to determine lysine content in *F. filiformis* [26,27]. The dried mycelium was ground into powder with a mortar. The mycelium powder (0.05 g) was accurately weighed, and 2 mL of deionized water was added prior to sonication for 2 h. Next, samples were centrifuged at 12,000 r/min for 10 min. The supernatant was the sample. Using another 2 mL centrifuge tube, we added 100 μL phosphate buffer with pH of 2, 200 μL 5.5 M glycol ether solution, 200 μL 0.08 M CuCl_2_ solution and 300 μL 0.06 M ninhydrin solution successively. Finally, the 100 μL sample was added to the mixture and reacted in boiling water for 35 min. The blank sample (deionized water) was also added to the mixture and reacted in boiling water for 35 min. The absorbance was determined using a spectrophotometer at a wavelength of 480 nm.

### 2.4. Optimization for Chitosan-Induced Lysine Accumulation in F. filiformis by Response Surface Methodology

The experimental data were analyzed using Design Expert 8.0 software. Central composite design (CCD) was adopted. The treatment time and concentration of chitosan were selected as independent variables (X) and lysine content as the dependent variable (Y). A two-factor and five-level response surface design was carried out to determine the optimal conditions for the lysine content of *F. filiformis* as induced by chitosan. Subsequent experiments were carried out according to the combination of these conditions to obtain the actual lysine content of *F. filiformis*. The difference between the actual lysine content and the predicted lysine content was analyzed.

### 2.5. RNA Extraction and Gene Expression Analysis

Total RNA was extracted from 25 mg of *F. filiformis* mycelium using 500 μL RNAiso Plus (TaKaRa, Dalian, China). A 5 × All-In-One RT MasterMix kit (TaKaRa, Dalian, China) was used to obtain cDNA. Then, quantitative real-time PCR was performed with Eppendorf Mastercycler ep Realplex 2.2 software (Eppendorf, Hamburg, Germany) [28]. The 18S rRNA of *F. filiformis* was used as the housekeeping gene. The 2^−ΔΔCT^ method was used to determine the relative expression levels of the genes [29].

### 2.6. Construction of an AAT-Silencing Vector and Transformation

Silent transformants were constructed using an RNA interference (RNAi)-mediated gene silencing strategy. The fungal RNAi vector pAN7-dual was used to construct *AAT*-silenced strains of *F. filiformis* [30]. Fragments of the *AAT* coding regions were amplified by PCR using *F. filiformis* cDNA as the template and the primers listed in Table S1. The PCR amplified fragment was connected to the PMD-19T vector. After successful sequencing, the *AAT3* and *AAT4* fragments were double-digested with the restriction enzymes KpnI and SpeI, then inserted into the pAN7-dual plasmid. Then, the *AAT3* and *AAT4* gene silencing plasmids (FF-AAT-RNAi) were transferred into *F. filiformis* via liposome-mediated transformation [31], and the sample was cultured at 25 °C for about two weeks. When the silencing transformants were selected, the gene expression levels of *AAT3* and *AAT4* were analyzed by quantitative real-time PCR. The *AAT*-silenced strains were called AATi. The empty vector control was named Si-control.

### 2.7. Statistical Analysis

Each statistical experiment was repeated at least 3 times independently. The experimental data were analyzed by IBM SPSS Statistics 25 (IBM, New York City, America) and plotted by GraphPad Prism 6. Each experimental datapoint shown in the graph is presented as mean ± SD. The different English letters in the graph indicate the significant difference between the different treatments; *p* < 0.05 is considered to be significant.

## 3. Results

### 3.1. Effects of Chitosan Treatment for Different Concentrations on Lysine and Protein Content of Mycelium of F. filiformis

**Figure 1:**
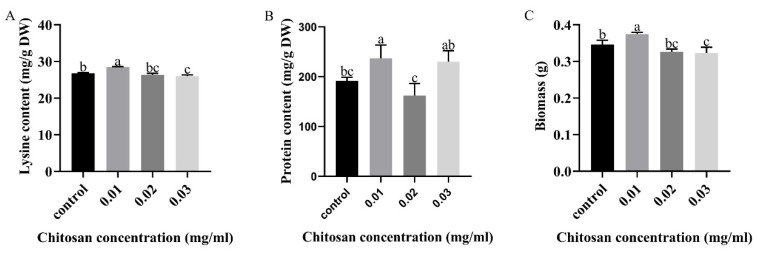
Effects of chitosan on (**A**) lysine, (**B**) protein and (**C**) biomass of *F. filiformis* mycelium. All calculated results are expressed as mean ± standard deviation, and different English letters indicate significant differences among different treatments (*p* < 0.05).

In order to study the effect of chitosan on lysine biosynthesis of *F. filiformis*, different concentrations of chitosan were added for induction. Lysine content in the mycelium of *F. filiformis* showed a trend of first rising then falling, and the lysine content increased by 6.42% with treatment with 0.01 mg/mL of chitosan (Figure 1A). At the same time, the contents of protein and mycelium biomass increased by 25.68% and 8.38%, respectively, in the treatment of 0.01 mg/mL of chitosan compared with the control (Figure 1B,C). The above results show that the contents of lysine, protein and biomass of *F. filiformis* mycelium were significantly increased with 0.01mg/mL chitosan treatment.

### 3.2. Effects of Chitosan Treatment Duration on Lysine and Protein Content of Mycelium of F. filiformis

**Figure 2:**
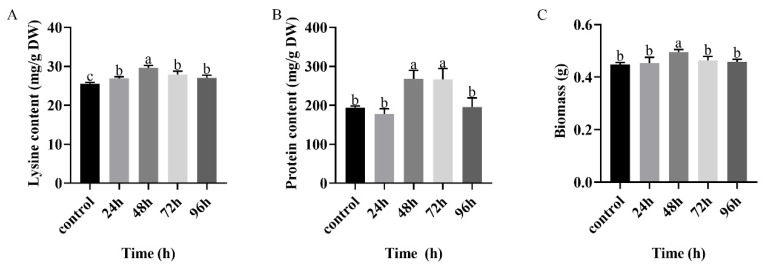
Effects of chitosan treatment of different durations on (**A**) lysine, (**B**) protein and (**C**) biomass of *F. filiformis* mycelium. All the calculated results are expressed in the form of mean ± standard deviation, while different English letters indicate significant differences among different treatments (*p* < 0.05).

In order to study the effects of chitosan treatment duration on lysine and protein contents of *F. filiformis*, 0.01 mg/mL chitosan was selected to treat *F. filiformis* for 0–96 h. The lysine content and biomass of *F. filiformis* showed a trend of first increasing and then decreasing (Figure 2). The maximum accumulation of lysine was 29.66 ± 0.70 mg/g, and the mycelium biomass also reached the maximum value of 0.496 ± 0.01 g when treated with chitosan for 48 h (Figure 2A,C). At the same time, protein content increased by 37.84% with treatment with 0.01 mg/mL chitosan for 48 h compared with the control (Figure 2B). Lysine content in *F. filiformis* increased with 0–96 h of chitosan incubation, and showed a trend of rising first then falling. The above results show that duration of chitosan treatment can significantly affect the lysine content of *F. filiformis*, and the induction has a time effect. The lysine, protein content and mycelium biomass of *F. filiformis* were increased significantly when treated with 0.01 mg/mL chitosan for 48 h.

### 3.3. Response Surface Methodology to Optimize Chitosan Treatment Conditions

The above test results show that the concentration and time of chitosan treatment can improve the lysine content of *F. filiformis*. Therefore, the central composite design (CCD) method was used to optimize the effects of chitosan treatment on the lysine content of *F. filiformis*. The treatment time and concentration of chitosan were selected as independent variables (X) and lysine content as dependent variable (Y). The experimental data were analyzed using Design Expert 8.0 software (StatEase, Minneapolis, America). The chitosan CCD test design is shown in Table 1, the response surface model analysis is shown in Table 2, and the regression analysis is shown in Table 3. The quadratic multiple regression equation obtained after software analysis is:

Y = 22.81 + 0.15 × Time + 0.58 × Concentration + 5.94 × 10^−4^ × Time × Concentration − 1.48 × 10^−3^ × Time^2^ − 0.02 × Concentration^2^.

In this equation, Y is the dependent variable representing lysine content; Time and Concentration are the time and concentration, respectively, of chitosan treatment. The results show that the F value of the model is 12.47, and the *p*-value (Prob > F) value is less than 0.0001, which means that the model is significant. The correlation coefficient R^2^ is greater than 0.8, and the coefficient of variation is 3.72%, which indicates that there is a good degree of fit between the actual value of the lysine content and the predicted value obtained by the software.

**Figure 3:**
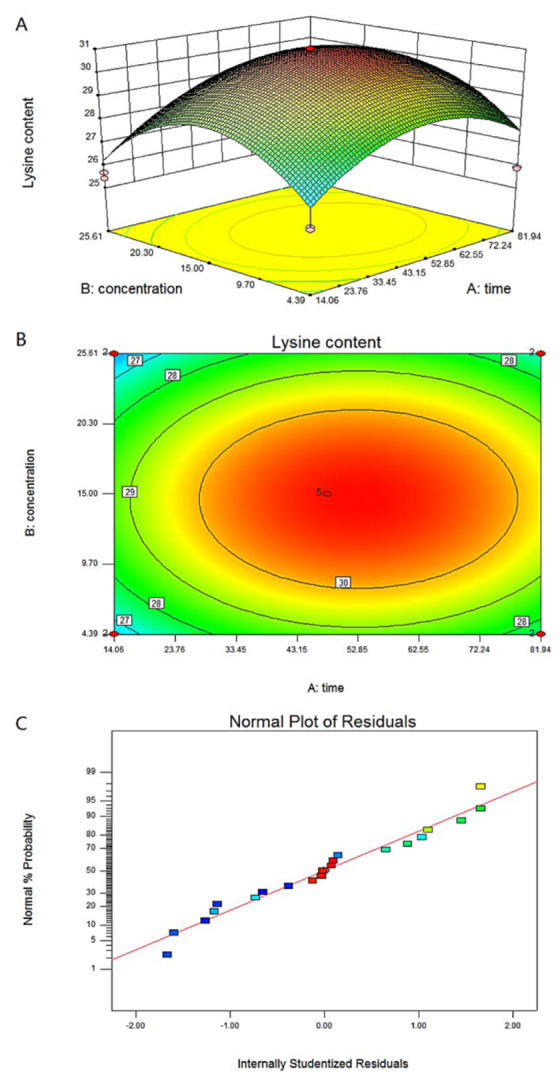
**Optimization of lysine accumulation conditions induced by chitosan in *F. filiformis*.** (**A**) A five-level two-factor center combination design is used to maximize the lysine content of *F. filiformis*. The independent variables are chitosan treatment concentration and treatment time. Table 1 shows the range and level of the independent variables. The levels of the variables are determined based on single factor analysis. (**B**) Contour plots reflect the combined effects of concentration and treatment time on lysine content in *F. filiformis*. (**C**) Normal probability plot of standardized residuals. Each color block is a response value.

The results show that the concentration and duration of chitosan treatment and their interaction all affect the lysine content of *F. filiformis* (Figure 3). Each point falls on almost a straight line, indicating that the result is reliable (Figure 3C). By combining and optimizing the two factors, according to the software analysis and optimization results: the concentration of chitosan is 14.61 μg/mL, the treatment time of chitosan is 52.90 h, and the predicted value of Y is 30.95 mg/g. Experimental verification was carried out according to the combination of the optimized experimental treatment conditions. The average of three parallel test results is 30.92 mg/g, which is not much different from the predicted value of 30.95 mg/g.

### 3.4. Chitosan Treatment Promotes the Growth of the Fruiting Body of F. filiformis

**Figure 4:**
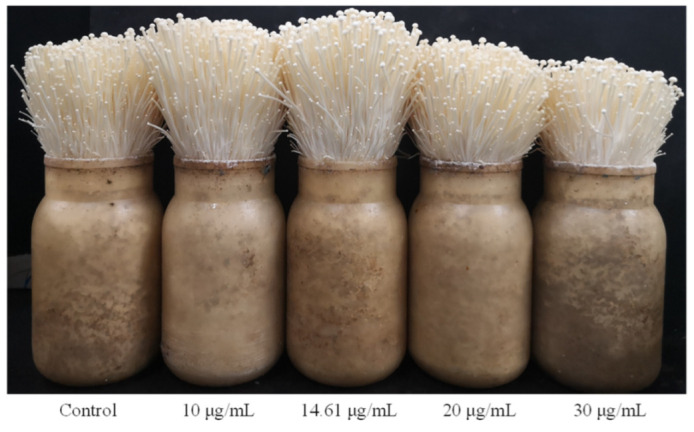
Fruiting body growth of *F. filiformis* treated with chitosan.

*F. filiformis* was inoculated aseptically and cultured in the factory for about 55 days. Sterile water was used in the control group, and 10 μg/mL, 14.61 μg/mL, 20 μg/mL and 30 μg/mL of chitosan were added for spraying treatment at the stage during which they needed to be watered during cultivation. The growth status of *F. filiformis* fruiting body that was treated with chitosan is shown in Figure 4. Compared with the control group, the growth rate of fruiting body of *F. filiformis* treated with 14.61 μg/mL chitosan increased by 23.35% (Table 4). The growth rate of *F. filiformis* was the fastest at this concentration. The results show that lysine content and the growth rate of fruiting body of *F. filiformis* could be increased by adding 14.61 μg/mL chitosan optimized by RSM. It was proven that optimization of the induction conditions of chitosan by RSM is very reliable. Therefore, chitosan can be added in the cultivation and production of *F. filiformis*.

### 3.5. Genetic Screening of Amino Acid Transporters Induced by Chitosan

**Figure 5:**
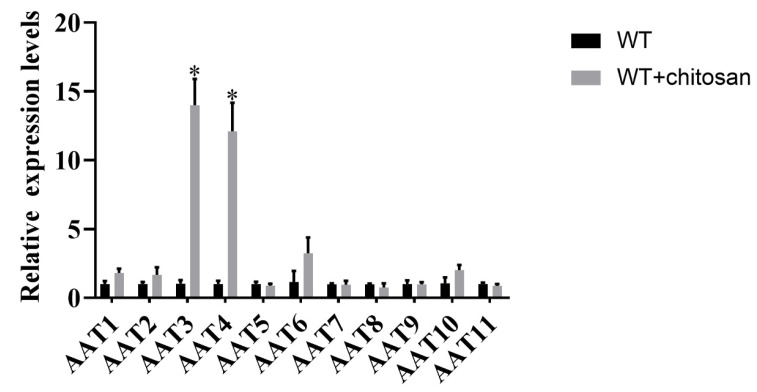
**Gene screening of amino acid transporters treated with chitosan.** The transcriptional levels of each *AAT* gene in WT strain were detected after chitosan treatment. The results are presented as mean ± standard deviation, and the asterisk indicates that there was a significant difference between the experimental group and the control group, * *p* < 0.01.

Amino acid transporters mediate the movement of amino acids into and out of cells or organelles [32]. To determine whether amino acid transporters are involved in the regulation of the biosynthesis of lysine induced by chitosan in *F. filiformis*, genetic screening of amino acid transporters induced by chitosan was carried out. Homologous sequence alignment was performed based on amino acid transporters in fungi that have been previously reported. Eleven homologous genes encoding amino acid transporters were found in *F. filiformis* genome (Table S2). These homologous proteins are named AAT1-AAT11. The expression levels of most genes did not change significantly with chitosan addition (Figure 5). The expression levels of *AAT3* and *AAT4* genes were significantly increased when chitosan was added. Transcription levels of AAT3 and AAT4 were increased 14-fold and 12-fold, respectively, compared with WT. The results showed that AAT3 and AAT4 responded to chitosan supplementation.

### 3.6. Construction of AAT3 and AAT4 Genes Silencing Strains of F. filiformis

**Figure 6:**
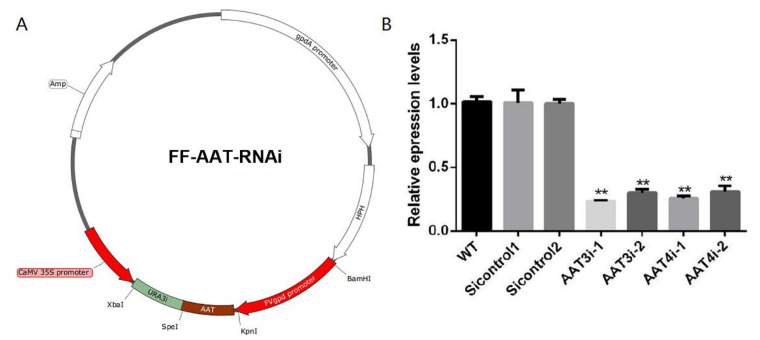
**Construction and screening of AAT3-silenced and AAT4-silenced strains.** (**A**) Construction of the *AAT3* and *AAT4* RNAi expression cassette plasmids. The *AAT3* and *AAT4* fragments were double-digested with the restriction enzymes KpnI and SpeI, then inserted into the pAN7-dual plasmid. In the plasmid, URA3 transcription is driven by the 35S promoter, and the *AAT* gene is driven by the glyceraldehyde-3-phosphate dehydrogenase promoter. (**B**) QRT-PCR analysis of the expression of *AAT* in the tested strains. The expression levels of the *AAT3* and *AAT4* genes in the WT strains were arbitrarily set to 1. The values are the mean ± standard deviations (SD) of the results of three independent experiments. The asterisks indicate significant differences compared with the untreated strains (Student’s *t*-test: ** *p* <0.01).

To further verify the roles of AAT3 and AAT4 in chitosan-induced lysine biosynthesis in *F. filiformis*, *AAT3* and *AAT4* gene silencing plasmids FF-AAT-RNAi were subsequently constructed (Figure 6A). When the proposed silencing transformants were selected, the gene expression levels of *AAT3* and *AAT4* were analyzed by quantitative real-time PCR to confirm the silencing efficiency of *AAT3* and *AAT4* gene-silencing strains. Subsequently, two *AAT3* gene silencing transformants (AAT3i-1 and AAT3i-2) with *AAT3* silencing efficiencies of about 76% and 70%, respectively, and two *AAT4* gene silencing transformants (AAT4i-1 and AAT4i-2) with *AAT4* silencing efficiencies of about 75% and 67%, respectively, were randomly selected for subsequent experiments (Figure 6B).

### 3.7. Changes to Lysine Content in AAT3 and AAT4 Silencing Strains under Optimal Chitosan Induction

**Figure 7:**
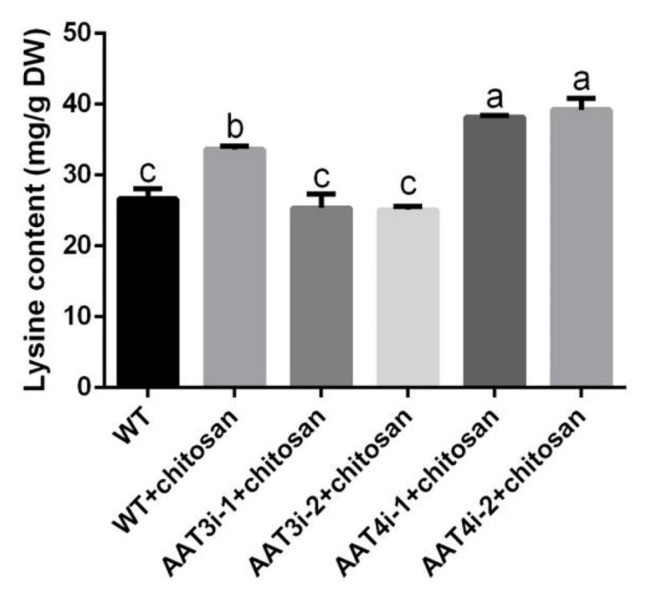
AAT3 and AAT4 were involved in the biosynthesis of lysine in *F. filiformis* with chitosan treatment. Lysine content in *F. filiformis* treated with chitosan was determined. The calculation results are presented as mean ± standard deviation, and different letters indicate significant differences between different treatments (*p* < 0.05).

The changes to lysine content in WT, AAT3i and AAT4i strains were further detected with treatment with chitosan. The results show that lysine content in the AAT3i strain decreased by 24.87% compared with the WT strain induced by chitosan. With chitosan treatment, the lysine content of the AAT4i strain was significantly increased by 13.55% compared with the WT strain (Figure 7).

## 4. Discussion

The cultivation of *F. filiformis* has been industrialized and automated. The previous work on industrial breeding of *F. filiformis* has been mainly for yield improvement up to now. There are few studies on improving the quality of *F. filiformis*. While focusing on high yield of *F. filiformis*, the production of high-quality *F. filiformis*, such as *F. filiformis* with high lysine content, can be used to enhance the competitiveness of *F. filiformis* in factory products. Therefore, in order to further change the production status of *F. filiformis*, higher requirements are put forward for the quality of *F. filiformis*. The two main conditions for the growth and development of edible fungi are nutrition characteristic conditions (carbon source, nitrogen source, inorganic salts, vitamins, etc.) and environmental conditions (temperature, moisture, air, light, pH, etc.) [33,34]. For *F. filiformis* factory production, the technology of improving *F. filiformis* yield by changing environmental factors is relatively mature. Adding exogenous inducers to influence the growth and development of edible fungi has more research value. The addition of exogenous salicylic acid to *Ganoderma lucidum* can significantly increase ganoderic acid content [35]. Spraying with chitosan significantly increased polyphenols in *Hericium erinaceus* by 42% [36]. Methyl jasmonate improved the appearance of *Agaricus bisporus* by inhibiting browning and color variation [37]. In this paper, improvement of lysine content in *F. filiformis* treated with chitosan was also observed. These findings show that exogenous inducers can effectively improve the nutritive quality of edible fungi.

Chitosan is a kind of natural alkaline polysaccharide without toxicity or pollution [38]. It comes from a wide range of sources and is widely used in food, agriculture and other fields [39]. In this paper, we found that chitosan treatment improved the quality of *F. filiformis*. Chitosan treatment significantly increased the content of curcumin, which is the main secondary metabolite of turmeric plants [40]. The application of chitosan, which is used to increase the concentration of bioactive compounds in broccoli sprouts, increased the content of vitamin C of 5-day-old broccoli sprouts by 54% [41]. Germination of pepper seeds treated with chitosan was increased under wet and cold conditions [42]. Chitosan is a proven exogenous inducer for improving quality in many crops. To summarize, chitosan has several industrially important characteristics such as low cost and low dosage/efficacy on several quality parameters, and it can be easily applied in a large-scale setting.

Amino acid transporters can transport amino acids into cells to participate in the synthesis of proteins, as well as play an indispensable role in biological growth and metabolism. At present, the three major superfamilies of AATs are mitochondrial carrier family (MCF), amino acid, polyamine and choline transporters (APC) and amino acid transporter family (ATF). The specific functions of AATs in organisms have also been gradually explored. Studies have shown that *Arabidopsis* under exogenous stress can increase the accumulation of proline through the mediation of amino acid permease 1 (AAP1) [43]. The content of free amino acids in an *Arabidopsis AAP8* mutant was greatly reduced [44]. These all indicate that AAT can affect the content of amino acids. In this study, we found that lysine content in *F. filiformis* was increased with chitosan induction. The expression levels of *AAT3* and *AAT4* genes were significantly increased with chitosan. It was speculated that AAT4 might play a role in the excretion of lysine when chitosan was added. After silencing the *AAT4* gene, lysine could not be effluxed, so lysine content was higher than that of the WT treated with chitosan. On the contrary, AAT3 might play a role in lysine absorption with chitosan treatment, and the strain could not absorb lysine after silencing the *AAT3* gene. The results suggest that AAT3 and AAT4 have different directions of lysine transport. The *Arabidopsis thaliana* bidirectional amino acid transporter 1 (BAT1) displays both export and import activity. BAT1 can regulate the inflow of arginine and alanine and the outflow of lysine and glutamate [45]. The lysine histidine transporter 1 is involved in *A. thaliana* uptake of amino acids [46]. In *Corynebacterium glutamicum*, SerE exhibited the ability to export L-threonine [47]. It can be seen that some AATs expel amino acids, some AATs absorb amino acids, and some AATs can both expel and absorb amino acids. These studies are consistent with our results. However, how AAT3 and AAT4 participate in chitosan-induced lysine biosynthesis in *F. filiformis* and whether they can influence lysine biosynthesis in *F. filiformis* through the interaction of other signal molecules needs further study.

## 5. Conclusions

In conclusion, this experiment proved that the addition of a certain concentration of chitosan can induce the accumulation of lysine in *F. filiformis*. It was further found that there are 11 members of AAT in *F. filiformis*. The expression levels of *AAT3* and *AAT4* genes increased significantly with chitosan treatment. The results indicate that AAT3 and AAT4 are involved in the regulation of the biosynthesis of lysine induced by chitosan in *F. filiformis*. AAT3 may play a role in in the absorption of lysine, and AAT4 may participate in the excretion of lysine with chitosan treatment.

## Figures and Tables

**Table 1:** Design matrix of CCD.

	Factor 1	Factor 2	Response 1
Run	A: Time (h)	B: Concentration (μg/mL)	Lysine Content (mg/g DW)
1	81.94	25.61	26.77
2	0.00	15.00	27.38
3	14.06	25.61	25.67
4	48.00	0.00	27.76
5	81.94	4.39	25.92
6	48.00	15.00	30.88
7	48.00	15.00	30.89
8	14.06	4.39	25.77
9	48.00	30.00	26.92
10	14.06	25.61	25.43
11	0.00	15.00	27.58
12	96.00	15.00	29.65
13	48.00	30.00	26.14
14	48.00	0.00	27.94
15	14.06	4.39	25.66
16	81.94	25.61	26.39
17	48.00	15.00	30.98
18	81.94	4.39	25.86
19	48.00	15.00	30.79
20	96.00	15.00	29.16
21	48.00	15.00	31

Note: There are 21 experimental combinations in this design. Each experiment was repeated three times to calculate the average lysine content of *F. filiformis*. The results were analyzed by second-order polynomial regression.

**Table 2:** ANOVA for response surface quadratic model.

Source	Sum of Squares	DF	Mean Squares	F Value	*p*-Value (Prob > F)
Model	69.77	5	13.95	12.47	<0.0001
Residual	16.78	15	1.12		
Lack of fit	16.18	3	5.39	108.24	<0.0001
Pure error	0.60	12	0.050		
Cor total	86.55	20			

**Table 3:** The least-squares fit and coefficient estimate.

Factor	Coefficient Estimate	Standard Error	%95 CI Low	%95 CI High	F Value	*p*-Value (Prob > F)
Intercept	30.91	0.46	29.90	31.92		
A-Time	0.49	0.26	−0.073	1.05	3.45	0.0831
B-Concentration	−0.17	0.26	−0.73	0.40	0.40	0.5354
AB	0.21	0.37	−0.58	1.01	0.33	0.5760
A^2^	−1.70	0.32	−2.41	−1.00	26.78	0.0001
B^2^	−2.33	0.32	−3.03	−1.63	50.08	<0.0001

Note: A refers to time. B refers to concentration.

**Table 4:** Effects of different concentrations of chitosan on lysine content and growth rate in fruiting bodies of *F. filiformis*.

Chitosan Concentration	Average Lysine Content (mg/g)	Lysine Content Increase Ratio (%)	Average Growth Length (cm)	Growth Length Increase Ratio (%)
Control	9.58 ± 0.31 c	-	13.06 ± 0.25 c	-
10 μg/mL	10.46 ± 0.15 b	9.19	14.48 ± 0.19 b	10.87
14.61 μg/mL	11.05 ± 0.23 a	15.34	16.11 ± 0.11 a	23.35
20 μg/mL	9.61 ± 0.26 c	0.31	12.93 ± 0.18 d	−1.00
30 μg/mL	8.57 ± 0.29 d	−10.54	11.80 ± 0.16 e	−9.65

Note: Different letters in the table indicate significant differences between treatments (*p* < 0.05).

## Data Availability

Data is contained within the article or Supplementary Materials.

## Supplementary Materials

The following are available online at https://www.mdpi.com/article/10.3390/foods11142163/s1, Table S1: Primer sequences used in this paper, Table S2: Coding genes and protein characteristics of 11 members of the amino acid transporter family of *F. filiformis*.
