# Supplementary material for: Chitosan Increases Lysine Content through Amino Acid Transporters in Flammulina filiformis
Source: Foods. 2022 Jul 21;11(14):2163. doi: 10.3390/foods11142163 (PMC9325215; doi:10.3390/foods11142163)
Supplement: Supplementary file 1 [file foods-11-02163-s001.zip › foods-1803530-supplementary.pdf]

Table S1

Primer	Sequence (5′to 3′)	Description
RT-18S-F	CTCGCTTTCCGTGTCCCTA	Detects the 18S expression
RT-18S-R	CATGGCAGCCTTGATCTCC	
RT-AAT1-F	TACTGCTGGAACCGAACT	Detects the AAT1 expression
RT-AAT1-R	ATGATTGTGAGGGATGTGA	
RT-AAT2-F	GACGCTTGTCGGTAT	Detects the AAT2 expression
RT-AAT2-R	GTGCTTTGCCCTCC	
RT-AAT3-F	GTTCATCGTGGCGGTATTCT	Detects the AAT3 expression
RT-AAT3-R	GCCCTCACAAACCTCGTCTT	
RT-AAT4-F	GAAGACAACTCGCAAAGG	Detects the AAT4 expression
RT-AAT4-R	GCATAGAACCGAAGATAGG	
RT-AAT5-F	GGTCTTGCCTCCATCTGT	Detects the AAT5 expression
RT-AAT5-R	CACCTTCTGCGTTTGCTC	
RT-AAT6-F	AAGACGAGCCACATACCA	Detects the AAT6 expression
RT-AAT6-R	TCCAGACCCGAGAAATAG	
RT-AAT7-F	CTTGGTTTCTGGGCTGTG	Detects the AAT7 expression
RT-AAT7-R	CATTGGATGCGATGAGGA	
RT-AAT8-F	TTTTGGAAATGAGCACCC	Detects the AAT8 expression
RT-AAT8-R	AAGTTGTTAGGCGATAGAAT	
RT-AAT9-F	ATACTGACTTCGGTCCTCT	Detects the AAT9 expression
RT-AAT9-R	GACACTGACACTGCTGGT	
RT-AAT10-F	GAAATGGTCACCTGGATA	Detects the AAT10 expression
RT-AAT10-R	GAGGATGGCAAGAAAGA	
RT-AAT11-F	GGGTATCACAGTTGGCTAT	Detects the AAT11 expression
RT-AAT11-R	CGAACGGGAAGGAAGT	
AAT3i-F	ACTGGGTACCTTATCGGGACAGGC	Detects the AAT3 expression
AAT3i-R	ACTGACTAGTCAAGATGATGAGACCAA	
AAT4i-F	ACTGGGTACCTTATTGCTCGGTTATG	Detects the AAT4 expression
AAT4i-R	ACTGACTAGTCAAATGGACCTGGAT	

Table S2 Coding genes and protein characteristics of 11 members of the amino acid transporter family of *F. filiformis*

ID	Genome sequence number	Gene length (bp)	cDNA length (bp)	The number of amino acid residues	Theoretical Isoelectric Point	Molecular weight (kD)
AAT1	Fv5446	2551	1767	588	7.90	61.28
AAT2	Fv7531	6122	5796	1931	6.47	211.60
AAT3	Fv2661	2023	1614	537	9.16	58.59
AAT4	Fv2311	3056	2467	822	9.74	90.81
AAT5	Fv5457	1930	1623	540	8.06	59.33
AAT6	Fv1945	2234	1740	579	6.67	63.45
AAT7	Fv2958	2081	1665	554	7.11	59.89
AAT8	Fv7212	2193	1614	537	8.24	58.03
AAT9	Fv181	2762	2634	877	8.39	97.83
AAT10	Fv894	1949	1524	507	9.34	56.22
AAT11	Fv8268	1326	732	243	6.81	26.46
